# Programmable Dynamics of Exchange‐Biased Domain Wall via Spin‐Current‐Induced Antiferromagnet Switching

**DOI:** 10.1002/advs.202100908

**Published:** 2021-07-15

**Authors:** Hyun‐Joong Kim, Soong‐Geun Je, Kyoung‐Woong Moon, Won‐Chang Choi, Seungmo Yang, Changsoo Kim, Bao Xuan Tran, Chanyong Hwang, Jung‐Il Hong

**Affiliations:** ^1^ Quantum Technology Institute Korea Research Institute of Standards and Science (KRISS) 267 Gajeong‐ro Daejeon 34113 Republic of Korea; ^2^ Department of Physics Chonnam National University 77 Yongbong‐ro Gwangju 61186 Republic of Korea; ^3^ Department of Emerging Materials Science Daegu Gyeongbuk Institute of Science and Technology (DGIST) 333 Techno jungang‐daero Daegu 42988 Republic of Korea

**Keywords:** antiferromagnet switching, Dzyaloshinskii–Moriya interaction, exchange bias, magnetic domain wall motion, spin‐Hall current

## Abstract

Magnetic domain wall (DW) motion in perpendicularly magnetized materials is drawing increased attention due to the prospect of new type of information storage devices, such as racetrack memory. To augment the functionalities of DW motion‐based devices, it is essential to improve controllability over the DW motion. Other than electric current, which is known to induce unidirectional shifting of a train of DWs, an application of in‐plane magnetic field also enables the control of DW dynamics by rotating the DW magnetization and consequently modulating the inherited chiral DW structure. Applying an external bias field, however, is not a viable approach for the miniaturization of the devices as the external field acts globally. Here, the programmable exchange‐coupled DW motion in the antiferromagnet (AFM)/ferromagnet (FM) system is demonstrated, where the role of an external in‐plane field is replaced by the exchange bias field from AFM layer, enabling the external field‐free modulations of DW motions. Interestingly, the direction of the exchange bias field can also be reconfigured by simply injecting spin currents through the device, enabling electrical and programmable operations of the device. Furthermore, the result inspires a prototype DW motion‐based device based on the AFM/FM heterostructure, that could be easily integrated in logic devices.

## Graphical abstract

The role of magnetic field in the ferromagnetic domain‐wall (DW) motions within a micropatterned magnetic layer can be efficiently overtaken by the effective field from adjacent exchange‐coupled antiferromagnet. Using the spin structure of antiferromagnet controllable with spin current, highly adjusted motion of exchange‐coupled DW is demonstrated showing a prospect of field‐free electrical operations of DW‐based spintronics devices.

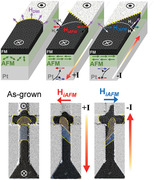

## 1 Introduction

Combining antiferromagnet (AFM) and ferromagnet (FM) adds versatile functionalities to spintronic devices and related technologies. In the AFM/FM heterostructure systems, FM spins, being coupled with adjacent AFM spins, tend to form an alignment different from a conventional ferromagnetic state in consistence with the AFM spin states close to the interface.^[^ ^1^, ^2^ ^]^ Phenomenologically, this effect appears as the presence of an effective internal magnetic field, commonly referred as exchange bias effect.^[^ ^1^, ^2^ ^]^ Exchange bias has conventionally been utilized to pin FM reference layers in recording heads or magnetic random‐access memory.^[^ ^3^, ^4^ ^]^ Very recently, exchange bias are being exploited in many spintronics device applications to implement the novel concepts of magnetic skyrmion^[^ ^5^, ^6^ ^]^ and field‐free spin‐orbit torque switchings.^[^ ^7^, ^8^ ^]^ In these examples, external magnetic fields (H_ext_), which normally acts globally with respect to the system dimensions, are replaced by localized exchange bias.

In the meantime, it is noted that the exchange bias can also be considered for an effective route to manipulate magnetic domain wall (DW) dynamics. In an ultrathin perpendicular magnetic system with broken structural inversion symmetry, the asymmetric exchange interaction, so called Dzyaloshinskii–Moriya interaction (DMI), is known to give rise to an in‐plane effective field H_DMI_ on the DW.^[^ ^9^, ^10^ ^]^ Therefore, it has received extensive attention due to the key role it can play in the formation of stable chiral DW and magnetic skyrmions.^[^ ^11^, ^12^, ^13^, ^14^ ^]^ The presence of DMI also introduces additional possibilities into the dynamics of DW, i.e., asymmetric and/or tilted DW motions. Both experimental^[^ ^15^, ^16^, ^17^, ^18^, ^19^ ^]^ and theoretical^[^ ^20^, ^21^ ^]^ works show that the asymmetric or tilted DW propagations arise from the modulation of DW structure which is stabilized by the Zeeman interactions between the DMI‐preferred chiral DW and an in‐plane H_ext_. Such method, however, may not be scalable for practical applications. In this regard, substituting the in‐plane H_ext_ with exchange bias field would be advantageous for the integration of DW motion based‐devices into electrical circuits. Despite such prospects, however, combining the exchange bias and DW dynamics has not been sufficiently explored yet.

We hereby investigate the manipulation of field‐driven DW motion in ultrathin perpendicular magnetic anisotropy (PMA) layer which is exchange coupled with adjacent AFM IrMn layer. In general, simultaneous application of both in‐plane and out‐of‐plane fields on the PMA FM layer is required in order to induce asymmetric DW motion.^[^ ^15^, ^16^ ^]^ In the present work, however, propagation of asymmetric DW was realized without applying an in‐plane H_ext_, which is effectively replaced by the exchange bias. In particular, the direction of the exchange bias is controlled by the polarity of a current pulse injected through AFM layer, realizing the control of DW tilt directions. Finally, we show that the programmable imprinting of DW propagation pattern at a cross‐bar device, which could be adopted to magnetic DW logic devices.

## 2 Results and Discussion

### 2.1 Existence of DMI in the Exchange Coupled HM/FM/AFM Trilayers

**Figure 1:**
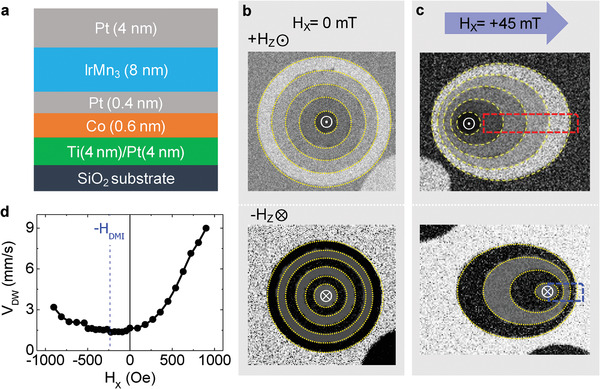
a) Schematic view of continuous multilayer. The exchange coupled HM/FM/AFM trilayers consisting of Pt (4 nm)/Co (0.6 nm)/Pt (0.4 nm)/IrMn_3_ (8 nm) were grown on Ti (4 nm) buffer layer on the SiO_2_ substrate with a Pt (4 nm) capping layer. b) MOKE images of up domain propagation (top panel) and down domain propagation (bottom panel) without H_X_. c) Propagations of up and down domains (top and bottom, respectively) in the presence of the in‐plane field bias H_X_ = +45 mT. d) DW speed (*υ*_DW_) of the DW in the red box as a function of H_X_ under the application of constant H_Z_ (±30 mT).

Multilayers of Ti (4 nm)/Pt (4 nm)/Co (0.6 nm)/Pt (0.4 nm)/IrMn_3_ (8 nm)/Pt (4 nm) on SiO_2_ substrate were deposited at room temperature by magnetron sputtering as illustrated in **Figure** **1**a. Depositions were carried out without H_ext_ to establish randomly distributed orientations of AFM domains in the initial as‐deposited state of the film. To confirm the existence of DMI in the deposited multilayers, the circular domain expansion with application of H_Z_ and H_X_ was examined using a magneto‐optical Kerr effect (MOKE) microscope.^[^ ^15^ ^]^ First, successive expansions of circular DW by repeated application of pulsed ±H_Z_ (+30 mT and −30 mT of H_Z_ for the top and bottom images, respectively) were measured and overlaid together as shown in Figure 1b. The circular DW expansion under the application of H_Z_ without H_X_ is clearly symmetric. On the other hand, Figure 1c shows asymmetric DW expansion under the pulsed ±H_Z_ with H_X_ bias of +45 mT. This asymmetric DW motion originates from different configurations between the in‐plane magnetic field H_X_ and H_DMI_.

Depending on whether the H_X_ is parallel (red dotted box) or antiparallel (blue dotted box) to the Néel‐wall component induced by H_DMI_, the DW motion can be regarded to be assisted or disturbed, respectively. Figure 1d displays the speed of DW (*υ*_DW_) in the red box with respect to applied H_X_. According to Je et al.,^[^ ^15^ ^]^ the DW energy (*σ*_DW_) in the presence of applied H_X_ and H_DMI_ is expressed by

(1) $$\sigma_{\mathrm{DW}}\left(H_{X}\right)=\sigma_{0}+2K_{D}\lambda -\pi \lambda M_{S}\cdot \left|H_{X}+H_{\mathrm{DMI}}\right|$$

where *σ*_0_ is the Bloch‐type DW energy density, K_D_ is the anisotropy energy density of DW, and *λ* is the DW width. In the DW creep regime,^[^ ^22^ ^]^ greater *σ*_DW_ results in lower *υ*_DW_. The maximum of *σ*_DW_ occurs when |H_X_ + H_DMI_| = 0, meaning that H_DMI_ is compensated by H_X_. In Figure 1d, the minimum of *υ*_DW_ is observed at H_X_ of approximately −25 mT, indicating that H_DMI_ is around 25 mT in the red dotted box, also confirming the existence of DMI in the deposited multilayers.

### 2.2 Tunable Asymmetric DW Motion via Reconfiguration of AFM Spins

**Figure 2:**
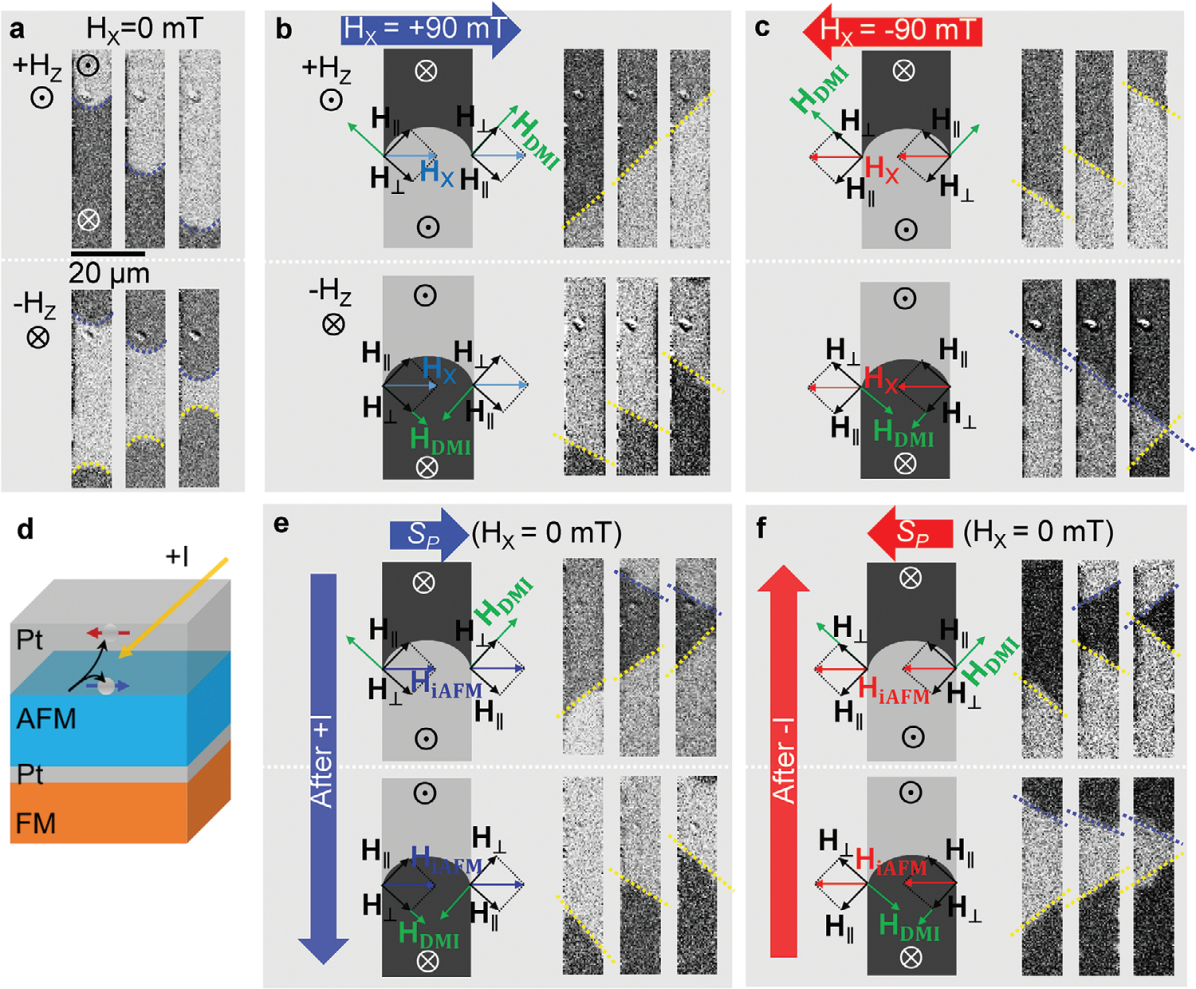
a) Up domain (top) and down domain (bottom) propagations in a wire‐patterned sample, intially in the as‐deposited state, without H_X_ bias. For all images in this figure, top panel (bottom panel) displays up (down) domain motion driven by +H_Z_ (−H_Z_). b,c) Schematic diagram of magnetic fields acting on DW and experimental DW propagation in the presence of ±H_X_. d) The schemetic of the injection of spin current, generated from top Pt layer, into AFM IrMn layer for an alignment of AFM spins. e,f) Schematic diagram of magnetic fields acting on the DW and the experimental DW propagations after the onset of ±H_iAFM_ by the eletric pulse injections. DWs with upward and downward motions were marked by yellow and blue dotted lines, repectively.

DW propagation was then investigated in the 10 µm‐width wire patterns as shown in **Figure** **2**. It is generally known that the DW in a wire‐patterned sample typically propagates as circular arc‐shape due to the pinning effect along both edges of the wire.^[^ ^22^, ^23^ ^]^ Figure 2a shows propagation of DW (top: up domain propagation, bottom: down domain propagation) by the pulsed ±H_Z_ ( = ±30 mT) without an application of H_X_. The DWs move with an arc‐shape having nearly identical displacements at both edges. As demonstrated by the asymmetric DW motion in Figure 1, the *υ*_DW_ can be modulated with the change of domain wall energy due to the H_X_ application in combination with DMI effect. Thus, we investigate the DW motion with simultaneous application of both H_Z_ and H_X_. Figure 2b,c shows the images of DW propagations by H_Z_ ( = ±30 mT) for H_X_ of +90 and −90 mT, respectively. Different from the case of H_X_ = 0 mT (Figure 2a), the application of H_X_ results in tilted DW propagation and the tilt angles are opposite for opposite directions of H_X_. The schematic diagrams in Figure 2b,c illustrates why the DW moves with tilt angle. H_X_ is decomposed into two orthogonal components, perpendicular (H_⊥_) and parallel (H_∥_), with respect to DW segment.

Domain wall speed, *υ*_DW_ varies depending on the relative configurations of H_⊥_ and H_DMI_ at a specific DW segment, as formulated in Equation (1) with the H_X_ substituted by H_⊥_ in the present case. The *υ*_DW_ is greatest when the H_⊥_ is parallel to H_DMI_, and lowest when H_⊥_ and H_DMI_ are antiparallel. Consequently, with the application of positive H_X_ (+90 mT) bias, the up‐down (from up domain to down domain) DW speed at the right edge of the wire should be higher than the speed along the left edge in the upper images of Figure 2b, while the down‐up DW speed at the left edge is higher in the bottom images. On the other hand, DW propagation under the application of identical ±H_Z_ with negative H_X_ (−90 mT) bias in Figure 2c shows opposite behavior with the fast up‐down DW speed at the left edge and the fast down‐up DW speed at the right edge. These asymmetric behaviors of tilted DW are in good agreement with the previous investigation by Kim et al.,^[^ ^16^ ^]^ where a crucial role of DMI for the asymmetric response of DW was pointed out.

It is notable that the FM layer in the multilayers has been exchange coupled to the adjacent AFM layer, but the coupling did not influence the dynamics of FM domains as the AFM layer is composed of many AFM domains with their Néel axes oriented randomly. Then the AFM spin alignment at the interface between FM Co layer and AFM IrMn layer was introduced to influence the motion of tilted DW. It is particularly conceivable that the AFM spin alignment might alleviate the need for application of external H_X_. Furthermore, there are increasing number of recent reports on the improved manipulations of local magnetic ordering state of AFM material employing the applications of electric current,^[^ ^24^, ^25^, ^26^, ^27^, ^28^, ^29^ ^]^ mechanical strain,^[^ ^30^, ^31^ ^]^ optical light with helicity,^[^ ^32^ ^]^ and electric field.^[^ ^33^, ^34^ ^]^ Recent demonstration of AFM Néel vector switching by current‐induced spin‐orbit torque (SOT) in various AFM systems^[^ ^35^ ^]^ has received increased attentions, and been found to be particularly interesting as well as useful. It was reported that the electrical manipulating Néel order of AFM by SOT could be realized through two possible mechanisms attributed to field‐like torque in AFM CuMnAs^[^ ^24^ ^]^ and Mn_2_Au,^[^ ^36^ ^]^ and damping‐like torque exerted by the polarized spin sources from heavy metal (HM) Pt layer due to a spin Hall effect (SHE) in Pt/AFM bilayers system such as Pt/CoO,^[^ ^37^ ^]^ Pt/NiO,^[^ ^38^, ^39^ ^]^ Pt/*α*‐Fe_2_O_3_,^[^ ^40^ ^]^ Pt/Mn_2_Au,^[^ ^41^ ^]^ and Pt/Mn_3_Sn.^[^ ^42^ ^]^ In the present works, spin current from HM Pt layer was injected to the AFM IrMn_3_ layer for the realignment of AFM spins as validated previously.^[^ ^27^ ^]^ Considering a much higher resistivity of IrMn (269.3 µΩ ⋅ cm)^[^ ^43^ ^]^ than that of Pt (17.3 µΩ ⋅ cm),^[^ ^44^ ^]^ applied current should mainly flow through the Pt layer and SHE in Pt is expected to play a dominant role over the presence of SHE in IrMn.^[^ ^45^ ^]^ Following an analogous procedure of flowing the charge current through the neighboring Pt layer directing the polarized spin Hall current to the adjacent IrMn AFM layer as illustrated in Figure 2d, proper AFM ordering of IrMn spins was achieved to exert an effective in‐plane exchange bias field on the FM layer. The polarized spin, **S_P_**, is directed normal to the charge current as represented by **S**_**P**_ ∝ **I** × **q**,^[^ ^46^ ^]^ where **I** and **q** represent charge and spin current flows, respectively. The damping‐like torque can be expressed as ***τ***  ∝  **m**  ×  (**m ** ×  **S**_**P**_), where **m** is magnetic moment vector of the AFM spin. Thus, individual magnetic moments in the AFM can be turned by the exerted spin torques, ***τ***, to align all spins into a uniformly ordered AFM state. Figure 2e,f shows perpendicular field‐driven DW propagation without application of H_X_ after positive and negative currents (±I) were flown along the length of the patterned wire. Surprisingly, after injecting a current pulse with ±45 mA‐amplitude and 1 ms‐length into the multilayers, the tilted DW propagation, which is usually activated by H_X_, is achieved without H_X_. We attribute this observation to the onset of H_iAFM_, the effective field due to the uniformly ordered AFM state of IrMn layer, that serves in place of H_X_ on the DW. The right‐polarized spins generated from Pt top layer were injected and exerted torque to turn the spins in the adjacent AFM IrMn layer. Thus, randomly distributed IrMn spins of the as‐grown state can be aligned uniformly along one transverse direction with respect to the wire due to the injection of right‐polarized spins. Consequently, the effective field, H_iAFM_ (represented as blue arrow in Figure 2e) due to a uniform ordering of AFM spins at the interface brings a change of typical arc shaped DW motion, implying that it plays the role of the applied external H_X_. The perpendicular and parallel components of H_iAFM_ (H_⊥_ and H_∥_) to DW segment are illustrated in Figure 2e. The perpendicular component of H_iAFM_ (H_⊥_) is along the same direction as H_DMI_ at the right edge and in opposite direction at the left edge, thereby the relationship between H_⊥_ and H_DMI_ leads to a local difference in DW energy. Figure 2e shows the propagation of tilted DW with high up‐down DW speed at right edge under +30 mT of H_Z_ (top three images) and high down‐up DW speed at the left edge under −30 mT of H_Z_ (bottom three images). Thus, we conclude that the tilted DW propagation is controlled by the uniform alignment of AFM spins only and note that the application of H_X_ is not necessary.

It is worth pointing out that the magnetic state of AFM does not change in the body of the AFM itself upon the 180° rotations of AFM Néel vector via the injection of either positive or negative polarized spin currents. However, exchange bias effect of the FM layer coupled to the adjacent AFM reflects the spin alignment state at the interface between AFM and FM, hence the switching of H_iAFM_ can be realized by 180° rotation of AFM Néel vector as well as the coupled uncompensated spins at the interface. Realignments of AFM spins at the interface in the opposite direction can also be readily achieved with the negative current pulse of −45 mA due to uncompensated interfacial AFM moments. The H_iAFM_ (red arrow in the illustration of Figure 2f), by realignment of AFM spins in the left direction after the injection of oppositely polarized spins, have an opposite effect on the DW motion in comparison to the H_iAFM_ (blue arrow in illustration of Figure 2e). As a result, Figure 2f shows opposite behavior of tilted DW propagation with fast up‐down DW speed at the left edge and fast down‐up DW speed at right edge with respect to the results in Figure 2e due to the H_⊥_ at the left edge aligned in the same direction as H_DMI_. It was apparently demonstrated that the uniform realignment of AFM spins through the injection of negative or positive spin Hall current can decide a tilt direction of the DW as it propagates along the line‐patterned magnetic track without an application of external transverse field, H_X_. In general, alignment of AFM spins for the exchange bias effect has conventionally been carried out by the field‐cooling method.^[^ ^1^ ^]^ While reliable, the conventional field‐cooling method may not be applicable to a multifunctional device with complex submicron scale structures because the direction of exchange bias is non‐switchable once it is set. In this regard, our electrical and switchable approach is particularly advantageous for the realization and repeated operations of the miniaturized DW motion‐based devices.

**Figure 3:**
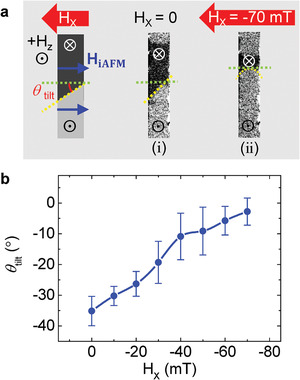
a) The image (i) shows the tilted DW motion (tilt angle, *θ*_tilt_) due to the action of H_iAFM_ and the image (ii) is flat DW motion where the H_iAFM_ is compensated by an additionally applied H_X_, resulting in zero net effective transverse field to the DW. b) Plot of the tilt angle (*θ*_tilt_) as a function of applied H_X_ bias is shown.

In order to quantify H_iAFM_, we investigated the DW propagation under an application of H_X_ bias field so that H_iAFM_ and H_X_ compete with each other. Initially, positive current pulse (+45 mA‐amplitude, 1 ms‐length) was injected into the wire, then the tilted DW with a tilt angle, *θ*_tilt_, was observed with an applied H_Z_ of +30 mT and zero‐H_X_ in the image (i) of **Figure** **3**a. The magnitude of *θ*_tilt_ is about 35˚ at zero‐H_X_. With the addition of H_X_, the tilt angle changes depending on the magnitude of H_X_. As shown in the DW propagation image (ii) of Figure 3a, it is remarkable that the *θ*_tilt_ can be set to nearly 0° under simultaneous application of H_X_ = −70 mT. The variation of *θ*_tilt_ during DW propagation driven by a constant H_Z_ was measured as a function of applied H_X_ ranging from 0 to −70 mT with a step of −10 mT. Figure 3b shows that the *θ*_tilt_ decreases gradually to zero as the magnitude of applied H_X_ increases. Therefore, we concluded that the H_iAFM_, induced by the aligned AFM spins, is effectively offset by the negative H_X_. From the H_X_ resulting in the zero tilt angle, the magnitude of H_iAFM_ can be deduced to be approximately 70 mT. Therefore, from the results of Figures 2 and 3, it is noteworthy that the uniformly aligned AFM spins within a plane, coupled to FM with DMI, successfully replaces the H_X,_ leading to the tilted DW motion. Additionally, to check whether the previous observations also hold for a system with the opposite DMI, we prepared multilayers of SiO_2_/Ti (4 nm)/Pt (4 nm)/IrMn_3_ (8 nm)/Pt (0.4 nm)/Co (0.6 nm)/Pt (2 nm), deposited in a reversed order (Figure S1a, Supporting Information) as compared to the previous multilayer stack. We find that the opposite DW tilt directions are observed for the same AFM setting currents (Figure S1b,c, Supporting Information), suggesting that our explanation for the H_iAFM_‐induced tilted DW motion also holds for the opposite DMI case.

### 2.3 Feasibility of Magnetic DW Logic Device

Recently, Luo et al.^[^ ^47^ ^]^ demonstrated that the chiral coupling between adjoining magnetic domains with competing DMI and the magnetic anisotropy could be exploited for the current‐driven DW logic circuits. Accordingly, in our present work, tunable asymmetric DW motion with competing DMI and unidirectional exchange anisotropies (H_DMI_ and H_iAFM_, respectively) should have a great potential to be utilized in the DW logic devices. In order to explore the feasibility with DW logic device, we present that the tilted DW propagation can be used to select its propagation path at the wire junctions or intersections by controlling the H_iAFM_.

**Figure 4:**
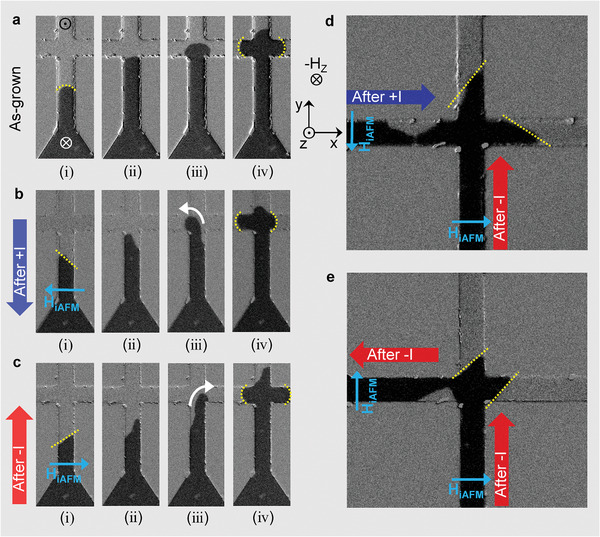
In the crossbar‐patterned sample, various behaviors of perpendicualr field driven down‐up DW motions without H_X_ can be generated dependening on the configuration of AFM spins. DW propagations were measured in a) as‐grown state with randomly distributed AFM domains, and after current injections of b) positive and c) negative currents along the vertical direction (*y*‐axis), respectively. DW motions in the horizontal branch after injection of d) positive current and e) negative current along *x*‐axis were also measured.

**Figure** **4** shows the propagation of tilted DW in the cross‐patterned film with identical layer structure to the one in Figure S1 (Supporting Information). Arc‐shaped down‐ up DW motion at a point of intersection was observed with the application of only negative H_Z_ in the as‐grown state of the sample without aligned AFM spins as shown in Figure 4a. Successive images (i) and (ii) in Figure 4a shows arc‐shaped DW without tilt before it arrives the intersection of the cross pattern. As it propagates through the junction, the DW trifurcate symmetrically into three channels and progress continuously at the same speed as shown in the images (iii) and (iv). On the other hand, when the AFM spins are aligned as in Figure 4b,c, the tilted DW propagations show asymmetric motion as it turn around corners of the intersection. In the case of AFM spin alignment with a positive current pulse (Figure 4b), successively taken images (i) and (ii) indicate clockwise‐tilt of down‐up DW. The DW speed is higher at the left edge of the wire, hence the DW turns early toward the left branch. Thus, the DW in the left channel propagated much faster than in the right channel, and the arrival time of the DW's at the branch ends are different. After the AFM spins have been realigned in opposite direction by injecting a negative current pulse, reversed DW tilt angle could be confirmed as mentioned previously, and the counterclockwise‐tilt of the down‐up DW is observed as shown in images (i) and (ii) of Figure 4c. Due to the opposite tilt of the DW front, as displayed in the images (iii) and (iv) of Figure 4c, the DW turns around the right corner easily and arrives at the right end earlier. Therefore, depending on the programed direction of H_iAFM_ by the current pulse polarity, the cross‐patterned device can allow DW to arrive at only one of the channels first, emulating logic operation.

Finally, we demonstrate that the DW propagation pattern in the cross‐patterned device can be imprinted by the combinations of H_iAFM_ setting the currents in *x* and *y* axes directions. Figure 4d,e displays typical examples of DW propagation patterns when the setting current combination are in (+*x*, −*y*) and (−*x*, −*y*), respectively. While the vertical propagation channels show the same DW tilt direction, the horizontal channels show the opposite DW tilt direction. This will result in additional selection of DW propagation when the horizontal channel encounters another intersection. In this cross‐patterned device, basically 4 different patterns can be programed. These results indicate the opportunity of controlling DW motions independently at a localized area within a complex patterned structure through a local and independent setting of H_iAFM_, in contrast to an inconvenient operation mechanism based on global application of external magnetic field. Furthermore, with the extension of the present study, controlled dynamics of asymmetric DW would be able to provide a novel mechanism to DW logic gates in spintronics devices comprised of complex patterns.

## 3 Conclusions

In summary, we introduced exchange coupling to the ultrathin PMA FM layer with DMI by placing an adjacent AFM layer. In the HM/FM/AFM trilayers, the behavior of perpendicular field‐driven DW motion was examined to find the modulation depending on the AFM spin configurations at the interface between AFM and FM layers. It was successfully demonstrated that the asymmetric DW propagates with a tilt angle controlled by an effective field of the uniformly aligned AFM spins (H_iAFM_) in combination with DMI field (H_DMI_) and that the control can be achieved by perpendicular field only without a need for in‐plane external magnetic field. Furthermore, the tilt angle of the DW front can be reliably reversed by the injection of spin current. Ultimately, it was shown that the propagation of tilted DW can be modulated or switched by the combined action of inherent H_DMI_ and spin current‐controlled H_iAFM_. Further development of the concept to combine the DMI and exchange bias for the controlled DW state as well as its dynamics is expected to bring an understanding of novel mechanism for the controlled SOT switching or manipulation of ultra‐small magnetic textures, that need to be resolved in relation with the recently raised issues on the field‐free SOT switching of perpendicular magnetization^[^ ^7^ ^]^ and stable skyrmion manipulations in exchange coupled AFM/FM structures,^[^ ^5^, ^6^ ^]^ respectively.

## 4 Experimental Section

Two types of multilayers consisting of Ti (4 nm)/Pt (4 nm)/Co (0.6 nm)/Pt (0.4 nm)/IrMn_3_ (8 nm)/Pt (4 nm) with positive sign of DMI and Ti (4 nm)/Pt (4 nm)/IrMn_3_ (8 nm)/Pt (0.4 nm)/Co (0.6 nm)/Pt (2 nm) with negative sign of DMI were deposited on SiO_2_ substrates at room temperature by magnetron sputtering with a base pressure of ≈1 × 10^−6^ torr. Deposition process was carried out without applying an external magnetic field on the substrate resulting in the randomly oriented AFM and FM magnetic domains. The wire and cross‐patterns with 10 µm width were manufactured by photolithography. Magnetic domains and the DW motions were observed by MOKE microscope. The duration of pulsed H_z_ was 100 ms in the DW motion experiments. H_X_ and H_Z_ were biased by using in‐plane and out‐of‐plane electromagnets, connected to the power supplies (BOP 20‐20DL and PBZ20‐20 manufactured by Kepco Inc. and Kikusui Electronics, Inc., respectively). In order to accurately align the H_X_ parallel to the film plane, the tilt sample stage was employed in the MOKE microscopy.

## Conflict of Interest

The authors declare no conflict of interest.

## Supporting information

Supporting Information

## Data Availability

Research data are not shared.
