# Supplementary material for: Programmable Dynamics of Exchange‐Biased Domain Wall via Spin‐Current‐Induced Antiferromagnet Switching
Source: Adv Sci (Weinh). 2021 Jul 15;8(17):2100908. doi: 10.1002/advs.202100908 (PMC8425944; doi:10.1002/advs.202100908)
Supplement: Supplementary file 1 — Supporting Information [file ADVS-8-2100908-s001.pdf]

WILEY-VCH

© 2021 Wiley-VCH GmbH

ADVANCED SCIENCE
Open Access

Supporting Information

for *Adv. Sci.*, DOI: 10.1002/advs.202100908

Programable dynamics of exchange-biased domain wall via spin-current-induced antiferromagnet switching

Hyun-Joong Kim, Soong-Geun Je, Kyoung-Woong Moon, Won-Chang Choi, Seungmo Yang, Changsoo Kim, Bao Xuan Tran, Chanyong Hwang, and Jung-Il Hong**

Programable dynamics of exchange-biased domain wall via spin-current-induced antiferromagnet switching

Hyun-Joong Kim, Soong-Geun Je, Kyoung-Woong Moon, Won-Chang Choi, Seungmo Yang, Changsoo Kim, Bao Xuan Tran, Chanyong Hwang, Jung-Il Hong**

Dr. H.-J. Kim, Dr. K.-W. Moon, Dr. S. Yang, Dr. C. Kim, Dr. C. Hwang
Quantum Technology Institute
Korea Research Institute of Standards and Science (KRISS)
267 Gajeong-ro, Daejeon 34113, Republic of Korea
E-mail: cyhwang@kriss.re.kr

W.-C. Choi, B. X. Tran, Prof. J.-I. Hong
Department of Emerging Materials Science
Daegu Gyeongbuk Institute of Science and Technology (DGIST)
333 Techno jungang-daero, Daegu 42988, Republic of Korea
E-mail: jihong@dgist.ac.kr

Prof. S.-G. Je
Department of Physics
Chonnam National University
77 Yongbong-ro, Gwangju 61186, Republic of Korea

Dzyaloshinskii-Moriya Interaction (DMI) in the multilayers studied in the present study is originated from the asymmetric arrangement of the layers. In order to confirm the role of DMI on the behavior of asymmetric DW motion in the multilayers system of SiO_2/Ti(4 nm)/Pt(4 nm)/ $IrMn_3$(8 nm)/Pt(0.4 nm)/Co(0.6 nm)/Pt(2 nm), the multilayers have been deposited in reverse order, as shown in Figure S1a, to obtain the DMI effect of opposite sign. When a negative current pulse (-45 mA amplitude and 1 msec width) is applied, polarized spins due to the spin Hall effect in the bottom Pt layer are injected into the adjacent IrMn AFM layer to align the AFM magnetic order, which then exerts exchange bias field to the FM layer. Figure S1b shows that the induced H_{iAFM} at the interface by uniform alignment of AFM spins with right direction is illustrated by blue arrow. When perpendicular component of H_{iAFM} ($H_{\perp}$) is parallel to negative H_{DMI} (purple arrow in Figure S1b), DW speed increases due to the reduced DW energy (σ_{DW}). Therefore, tilted DW with higher down-up DW speed at right

edge propagated under negative H_Z (-30 mT), which shows opposite behavior due to the negative H_{DMI} compared to the case in Figure 2e and Figure S1b with identical directions of H_{iAFM}. As the uniform alignment of AFM spins were reversed in opposite direction with a positive current pulse (+45 mA-amplitude and 1 msec-length) as shown in Figure S1c, tilted DW propagation exhibited opposite behavior corresponding to fast down-up DW motion at left edge. Therefore, the behavior of tilted DW is determined by a combination of the effective field (H_{iAFM}) due to the uniformly aligned AFM spins and the induced field (H_{DMI}) from the DMI effect

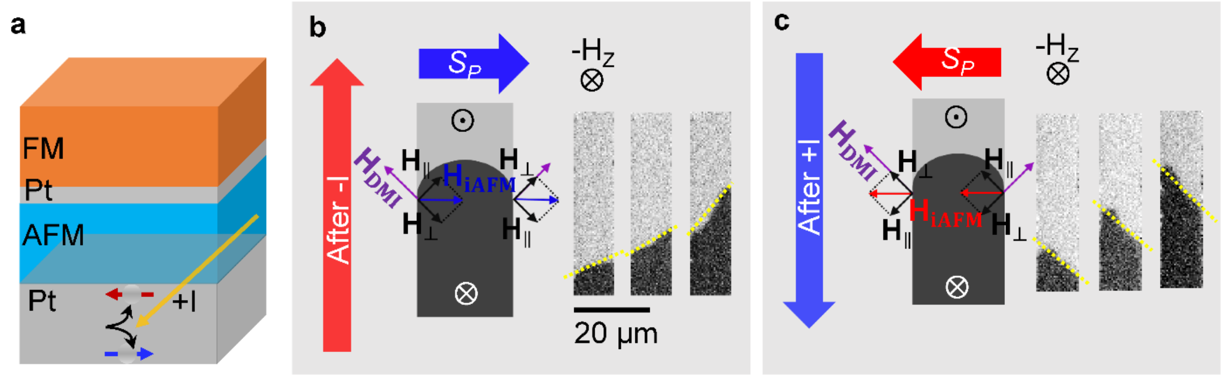

Figure S1. The exchange coupled HM/AFM/FM trilayers consisting of Pt(4 nm)/$IrMn_3$(8 nm)/Pt(0.4 nm)/ Co(0.6 nm) were grown on Ti (4 nm) buffer layer on SiO_2 substrate with capping layer of Pt (2 nm). a) The schemetic of the injection of spin current, generated from bottom Pt layer, into AFM IrMn layer for a uniform alignment AFM spins. In the case of uniformly aligned AFM spins, the puperdicular field driven down-up DW motion images after injection of b) negative current and c) positive current.
